# Supplementary material for: Impact of breast surgery on survival of patients with stage IV breast cancer: a SEER population-based propensity score matching analysis
Source: PeerJ. 2020 Mar 18;8:e8694. doi: 10.7717/peerj.8694 (PMC7085290; doi:10.7717/peerj.8694)
Supplement: Table S1 [file peerj-08-8694-s002.doc]

**Table S1:** Univariate Cox proportional hazard model for breast cancer-specific survival (BCSS) and overall survival (OS) in all patients with stage IV breast cancer.

| Variables | n | BCSS | | OS | |
| --- | --- | --- | --- | --- | --- |
|  |  | HR (95% CI) | *P* ^a^ | HR (95% CI) | *P* ^a^ |
| Age (years) | | | | | |
| 20-49 | 2999 | Reference |  | Reference |  |
| 50-79 | 10035 | 1.484 (1.402-1.571) | <0.001 | 1.534 (1.451-1.622) | <0.001 |
| Race | | | | | |
| White | 9588 | Reference |  | Reference |  |
| Black | 2331 | 1.309 (1.235-1.386) | <0.001 | 1.329 (1.257-1.405) | <0.001 |
| Others | 1061 | 0.908 (0.831-0.991) | 0.031 | 0.881 (0.808-0.960) | 0.004 |
| Unknown | 54 | 0.334 (0.174-0.642) | 0.001 | 0.312 (0.162-0.601) | <0.001 |
| T stage | | | | | |
| T1+T2 | 5578 | Reference |  | Reference |  |
| T3+T4 | 7456 | 1.395 (1.331-1.462) | <0.001 | 1.387 (1.326-1.451) | <0.001 |
| N stage | | | | | |
| N0+N1 | 9368 | Reference |  | Reference |  |
| N2+N3 | 3666 | 0.915 (0.870-0.963) | 0.001 | 0.915 (0.871-0.961) | <0.001 |
| Grade | | | | | |
| I+II | 5628 | Reference |  | Reference |  |
| III+IV | 6017 | 1.347 (1.283-1.414) | <0.001 | 1.313 (1.252-1.376) | <0.001 |
| Unknown | 1389 | 1.241 (1.148-1.341) | <0.001 | 1.242 (1.153-1.339) | <0.001 |
| Histology | | | | | |
| IDC | 8718 | Reference |  | Reference |  |
| ILC | 1154 | 0.981 (0.904-1.066) | 0.657 | 0.977 (0.902-1.059) | 0.573 |
| Others | 3162 | 1.411 (1.340-1.487) | <0.001 | 1.437 (1.366-1.511) | <0.001 |
| Molecular subtype | | | | | |
| HR+/HER2- | 6422 | Reference |  | Reference |  |
| HR+/HER2+ | 1982 | 0.768 (0.713-0.828) | <0.001 | 0.756 (0.703-0.813) | <0.001 |
| HR-/HER2+ | 1132 | 0.964 (0.882-1.054) | 0.424 | 0.950 (0.871-1.036) | 0.246 |
| TNBC | 1608 | 2.396 (2.246-2.557) | <0.001 | 2.318 (2.176-2.469) | <0.001 |
| Unknown | 1890 | 1.921 (1.803-2.048) | <0.001 | 1.933 (1.818-2.055) | <0.001 |
| Chemotherapy status | | | | | |
| Yes | 7462 | Reference |  | Reference |  |
| No/Unknown | 5572 | 1.548 (1.479-1.620) | <0.001 | 1.592 (1.524-1.664) | <0.001 |
| Radiation status | | | | | |
| No | 10692 | Reference |  | Reference |  |
| Yes | 2342 | 0.581 (0.545-0.619) | <0.001 | 0.568 (0.534-0.605) | <0.001 |
| Bone-only metastasis | | | | | |
| Yes | 4477 | Reference |  | Reference |  |
| No | 8557 | 1.681 (1.599-1.769) | <0.001 | 1.649 (1.571-1.732) | <0.001 |
| Surgery status | | | | | |
| No | 9151 | Reference |  | Reference |  |
| Yes | 3883 | 0.500 (0.474-0.527) | <0.001 | 0.500 (0.475-0.527) | <0.001 |

Abbreviation: HR, hazard ratio; CI, confidence interval; BCSS, breast cancer-specific survival; OS, overall survival; HR, hormone receptor; HER2, human epidermal growth factor receptor 2; TNBC, triple negative breast cancer.
