# Supplementary material for: Impact of breast surgery on survival of patients with stage IV breast cancer: a SEER population-based propensity score matching analysis
Source: PeerJ. 2020 Mar 18;8:e8694. doi: 10.7717/peerj.8694 (PMC7085290; doi:10.7717/peerj.8694)
Supplement: Table S2 [file peerj-08-8694-s003.doc]

**Table S2:** Univariate Cox proportional hazard model for breast cancer-specific survival (BCSS) and overall survival (OS) in 1:1 matched propensity score matching analysis with stage IV breast cancer.

| Variables | n | BCSS | | OS | |
| --- | --- | --- | --- | --- | --- |
|  |  | HR (95% CI) | *P* ^a^ | HR (95% CI) | *P* ^a^ |
| Age (years) | | | | | |
| 20-49 | 1204 | Reference |  | Reference |  |
| 50-79 | 3334 | 1.284 (1.171-1.408) | <0.001 | 1.344 (1.227-1.471) | <0.001 |
| Race | | | | | |
| White | 3284 | Reference |  | Reference |  |
| Black | 849 | 1.348 (1.222-1.486) | <0.001 | 1.368（1.245-1.503） | <0.001 |
| Others | 397 | 0.880 (0.756-1.025) | 0.100 | 0.871（0.751-1.010） | 0.068 |
| Unknown | 8 | 0.193 (0.027-1.370) | 0.100 | 0.182（0.026-1.290） | 0.088 |
| T stage | | | | | |
| T1+T2 | 2100 | Reference |  | Reference |  |
| T3+T4 | 2438 | 1.539（1.419-1.670） | <0.001 | 1.535（1.419-1.660） | <0.001 |
| N stage | | | | | |
| N0+N1 | 2884 | Reference |  | Reference |  |
| N2+N3 | 1654 | 1.141（1.052-1.238） | 0.002 | 1.147（1.060-1.241） | <0.001 |
| Grade | | | | | |
| I+II | 1674 | Reference |  | Reference |  |
| III+IV | 2583 | 1.773(1.619-1.941) | <0.001 | 1.691（1.550-1.845） | <0.001 |
| Unknown | 281 | 1.724(1.517-1.959) | <0.001 | 1.668（1.474-1.886） | 0.001 |
| Histology | | | | | |
| IDC | 3367 | Reference |  | Reference |  |
| ILC | 342 | 0.886(0.770-1.022) | 0.182 | 0.869（0.778-1.099） | 0.198 |
| Others | 829 | 1.100(0.994-1.218) | 0.066 | 1.116（0.092-1.231） | 0.085 |
| Molecular subtype | | | | | |
| HR+/HER2- | 2160 | Reference |  | Reference |  |
| HR+/HER2+ | 780 | 0.707(0.621-0.806) | <0.001 | 0.700（0.617-0.793） | <0.001 |
| HR-/HER2+ | 480 | 1.014 (0.878-1.172) | 0.848 | 1.006（0.875-1.156） | 0.935 |
| TNBC | 798 | 2.924 (2.647-3.229) | <0.001 | 2.808（2.549-3.093） | <0.001 |
| Unknown | 320 | 1.523 (1.312-1.767) | <0.001 | 1.516（1.313-1.749） | <0.001 |
| Chemotherapy status | | | | | |
| Yes | 3077 | Reference |  | Reference |  |
| No/Unknown | 1461 | 1.184 (1.089-1.287) | <0.001 | 1.220（1.125-1.322） | <0.001 |
| Radiation status | | | | | |
| No | 3695 | Reference |  | Reference |  |
| Yes | 843 | 0.939(0.848-1.039) | 0.224 | 0.911（0.824-1.006） | 0.065 |
| Bone-only metastasis | | | | | |
| Yes | 1548 | Reference |  | Reference |  |
| No | 2990 | 1.578 (1.445-1.723) | <0.001 | 1.546（1.420-1.682） | <0.001 |
| Surgery status | | | | | |
| No | 2269 | Reference |  | Reference |  |
| Yes | 2269 | 0.598(0.551-0.648) | <0.001 | 0.608（0.562-0.657） | <0.001 |

Abbreviation: HR, hazard ratio; CI, confidence interval; BCSS, breast cancer-specific survival; OS, overall survival; HR, hormone receptor; HER2, human epidermal growth factor receptor 2; TNBC, triple negative breast cancer.
